# In-cell chromatin structure by Cryo-FIB and Cryo-ET

**DOI:** 10.1016/j.sbi.2025.103060

**Published:** 2025-05-10

**Authors:** Zhen Hou, Peijun Zhang

**Affiliations:** 1 Division of Structural Biology, Nuffield Department of Medicine, University of Oxford, Oxford, OX3 7BN, UK; 2 Diamond Light Source, Harwell Science and Innovation Campus, Didcot, OX11 0DE, UK

## Abstract

Chromatin, the complex of DNA and proteins that organises genetic material in eukaryotic cells, has been a focal point of biological research for over a century. Its structure determines critical functions such as gene regulation, DNA replication and chromosome segregation. Early models of chromatin were limited by technological constraints, but advancements in imaging, particularly X-ray and electron microscopy (EM), gradually unveiled its hierarchical organisation. The recent emergence of cryo-electron tomography (cryo-ET) coupled with cryo-focused ion beam (cryo-FIB) milling has revolutionised our understanding of chromatin organisation by providing native, three-dimensional (3D) views of various macromolecules and architectures of chromatin at unprecedented resolution. This review traces the historical progression of chromatin structural studies, from early EM and fluorescence microscopy to the transformative insights offered by cryo-ET, culminating in a synthesis of current knowledge and future directions.

## Introduction

### The evolution of chromatin imaging

The human genome consists of approximately 3.2 billion base pairs and spans about 2 m in length [1]. How metres of DNA are efficiently packaged into microscale nuclei while remaining readily accessible when needed has been a long-standing question in science [2–5]. The 1974 nucleosome hypothesis by Kornberg posited DNA wrapped around histone octamers [3,4], later validated by the X-ray crystallography of nucleosomes, revealing atomic details [6,7]. Despite potential artefacts induced by chemical fixation and heavy metal staining [8], early EM studies revealed higher order of chromatin as fibrous networks, such as 10 nm ‘beads-on-a-string’ fibres, which further coil into 30 nm one-start solenoid or two-start zigzag fibres [9–17], confirming nucleosomes as the primary structural unit. Along the advancement of imaging techniques, questions arose about the prevalence of these models *in vivo* as critics argued that the 30 nm fibre was an artefact of sample preparation and isolation, as intact native nuclei rarely showed such regularity [18–20]. Cryo-EM mitigated some artefacts by imaging vitrified samples, yet grappled with chromatin’s heterogeneity, sample purification, *in vitro* reconstitution, and the challenge of imaging intact nuclei [21–24]. Fluorescence microscopy complemented cryo-EM by enabling dynamic, large-scale chromatin visualisation in living cells. Techniques like fluorescence *in situ* hybridisation (FISH) mapped specific loci, revealing chromosome territories and compartmentalisation [25,26]. Live-cell imaging with fluorophore-tagged histones or DNA-binding dyes showed chromatin motion during processes like mitosis and transcription [27,28]. However, fluorescence microscopy lacked molecular detail due to the limitations of resolution [29], being unable to resolve the conformations of nucleosomes and chromatin fibres. Therefore, the structure of native chromatin fibres remains elusive, underscoring the need for high-resolution *in situ*, or, in current parlance, in-cell imaging.

### Cryo-ET and cryo-FIB: a paradigm shifter in chromatin imaging

**Figure 1:**
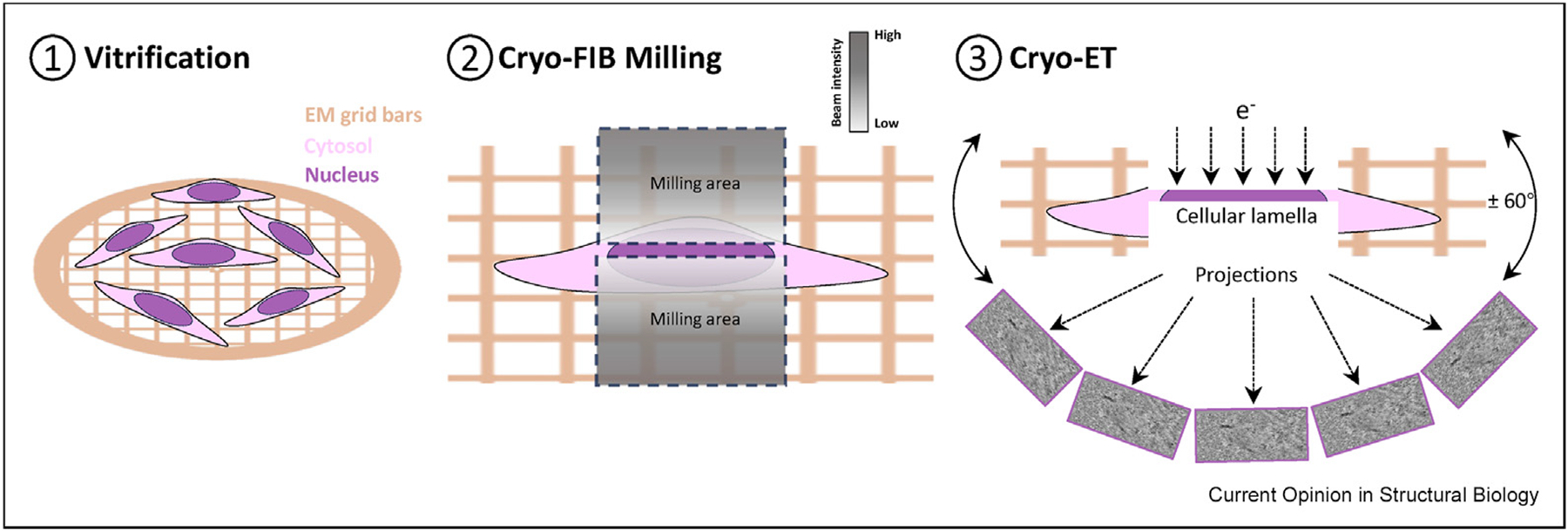
Workflow of cryo-FIB and cryo-ET in chromatin studies. Step one is to vitrify intact cells on the EM grid with optimal cell density. Step two is the preparation of cellular lamellae by cryo-FIB. Nuclei usually appear as humps on the EM grid. Stepwise milling, from high to low beam intensity is conducted parallelly above and below the region of interest (ROI), yielding a thin cell lamella (100–200 nm). Step three is the collection of a tilt series on the ROI in a cryo-TEM by tilting the grid symmetrically about 60°. Cryo-FIB, cryo-focused ion beam; cryo-ET, cryo-electron tomography; EM, electron microscopy.

Cryo-ET is an emerging technology that enables scientists to not only resolve the structure of large protein complexes (e.g. ribosomes, nuclear pore complexes, and viral particles) and those with preferred orientations or limited particle numbers, but also visualise native cellular contexts [30–34]. In cryo-ET, a biological sample is flash-frozen in vitreous ice to prevent dehydration and preserve structural integrity [34,35]. A series of tilt projection images are acquired by rotating the sample in a cryogenic transmission electron microscope (cryo-TEM). These images are computationally reconstructed into a tomogram, a 3D volume that reveals the organisation of macromolecular complexes at nanometre and even subnanometre resolution [36,37]. Moreover, when coupled with cryo-FIB, which is a state-of-the-art technology used to thin down native biological samples (e.g. intact cells and tissues) to lamellae with the thickness permitting the penetration of electrons, cryo-ET provides unrivalled opportunities to image macromolecules in their native environment in intact cells with molecular details (Figure 1) [36–47]. Notably, no chemical fixation or staining is required in the sample preparation of cryo-ET and cryo-FIB, and cellular lamellae prepared by cryo-FIB do not inherit any volume compression like cryosections [48]. Therefore, utilising cryo-ET combined with cryo-FIB to dissect the structure of native chromatin in the cell has spurred great interest in the past few years [38–40,44,46,47,49].

## Investigating higher orders of chromatin by *in vitro* cryo-EM and cryo-ET

The resolution revolution in cryo-EM [50] has encouraged significant efforts to study higher-order chromatin structures *in vitro* using cryo-EM and cryo-ET. These studies revealed chromatin in diverse forms, including short twisted fibres, large condensates formed by liquideliquid phase separation (LLPS), nucleosome stacks and long irregular fibres. In 2014, cryo-EM imaging of reconstituted chromatin identified a regular, short two-start tetranucleosomal fibre as a fundamental structural unit of chromatin [21]. A 2022 study leveraging cryo-ET demonstrated that manipulating the concentration of linker histone H1 drives the transition from isolated nucleosomes to condensed chromatin domains, underscoring H1’s critical role in chromatin organisation [51]. That same year, cryo-EM analysis of purified telomeres revealed chromatin fibres composed predominantly of nucleosome stacks and linear arrays, with no evidence of twisted two-start fibres [52].

Subsequent work on chromatin isolated from HeLa and K562 cells further demonstrated irregular two-start fibres organised into continuous and twisted structures [49,53]. Collectively, these studies resolve chromatin architecture at nanometre-scale resolution while capturing its conformational diversity. However, *in vitro* systems may lack cellular factors essential for native chromatin regulation, potentially introducing artefacts. This limitation necessitates investigations focused on chromatin within intact nuclei to elucidate its physiological organisation.

## In-cell structures of native chromatin and nucleosome by cryo-FIB and cryo-ET

**Figure 2:**
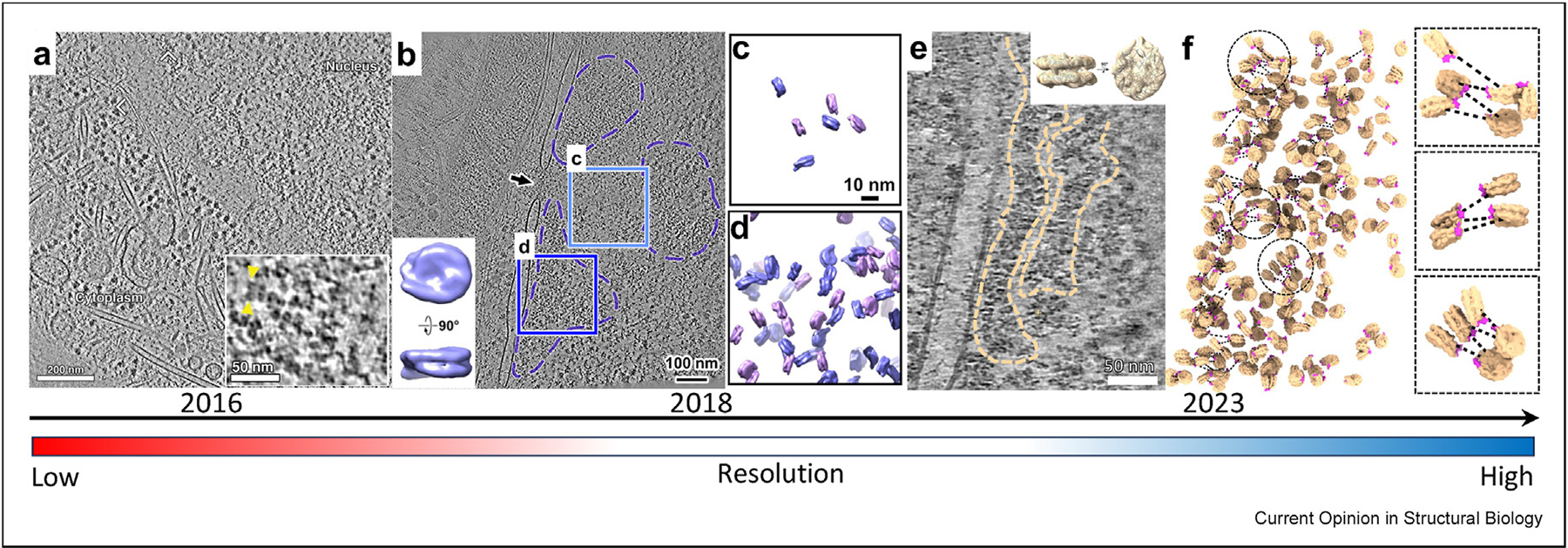
Chronological evolution of cryo-ET in-cell imaging of native chromatin. **(a)** A representative tomographic slice of an interphase Hela cell featuring the periphery of nucleus and cytoplasmic components. Chains of nucleosomes are showcased in the inset and individual nucleosomes are indicated by yellow arrowheads. Adapted from Ref. [39]. **(b)** A representative tomographic slice of an interphase Hela cell containing the periphery of nucleus and other cytoplasmic components. The black arrow indicates the nuclear pore complex, three dashed frames indicate three heterochromatic regions. Box c indicates a fraction of euchromatic regions and box d indicates a fraction of heterochromatic regions. The in-cell structure of native nucleosomes is depicted in the inset. **(c)** and **(d)**, show the mapping back of the two boxed regions in **(b)**. Adapted from Ref. [40]. **(e)** A representative tomographic slice of an interphase human T lymphoblast featuring two distinctive chromatin fibres in the heterochromatic region. The in-cell structure of native nucleosome is depicted in the inset fitted with a crystal structure of core nucleosome (PDB 6ESF). **(f)** Mapping back of individual nucleosomes in chromatin fibres in **(e)**. The H1 density is coloured magenta, indicating the side of entry and exit of the linker DNA, from which the DNA path (dashed lines) is predicted. Three representative subregions (circled) are enlarged on the right panel. Adapted from Ref. [41]. The bottom bar indicates the time points along with the increase in resolution of in-cell structures of nucleosomes. cryo-ET, cryo-electron tomography.

In 2013, scientists began investigating the structure of chromatin in intact cells of the picoplankton *Ostreococcus tauri* employing cryo-ETon cryosections, reminiscent of nucleosome particles could be identified, although with potential artefacts due to the volume compression from sectioning [54]. Later in 2016, advancements in cryo-ET combined with cryo-FIB milling enabled the direct visualisation of intact nuclei in human HeLa cells in great detail without perturbations. Individual nucleosomes were distinctly identified forming chains that could be traced, opening the possibility for *in situ* structural determination of nucleosomes and mapping them within the chromatin (Figure 2a) [38]. Closer to unveiling the ultrastructure of native chromatin, in 2018, individual nucleosomes of human Hela cells were characterised by template matching followed by subtomogram averaging (STA), yielding in-cell nucleosome structures at about 20 Å (Figure 2b) [39]. Through 3D classification, in-cell nucleosomes in the heterochromatin region could be divided into distinct groups featuring mono-, di- and tri-nucleosomes. Mapping back nucleosomes into the tomogram further revealed the chromatin domains in the intact nucleus (Figure 2c, d) [39]. However, tracing each nucleosome within the chromatin was still challenging due to the lack of resolution and no distinctive chromatin fibres were observed. In 2023, a landmark study leveraging cryo-ET and cryo-FIB on human T lymphoblast nuclei achieved unprecedented resolution (12Å) for in-cell nucleosome structures (Figure 2e) [40], approaching the subnanometre range. This advancement was enabled by generating ultra-thin lamellae (<100 nm), which produced tomograms with significantly enhanced signal-to-noise ratios (SNR). Strikingly, reconstructed tomograms revealed continuous chromatin fibres traversing heterochromatin domains near the nuclear envelope, with linker DNA discernible without computational enhancement (Figure 2e) [40]. Subsequent STA and 3D classification of template-matched nucleosomes identified two predominant mononucleosome populations: one with the linker histone H1 bound to the linker DNA and another devoid of H1 [40]. The revelation of H1 on the in-cell nucleosome structure led to the prediction of the trajectory between nucleosomes in the chromatin fibre, suggesting that native chromatin fibres adopt a flexible zigzag conformation, with nucleosomes connected irregularly (Figure 2f) [40], deviating from the canonical rigid 30 nm fibre model. These findings underscore chromatin’s flexibility, context-dependent compaction, challenging prior assumptions of uniformity and highlighting the heterogeneity in chromatinpacking nucleosomes.

## Disparities in native chromatin structures

The notion of a universal chromatin architecture across eukaryotes is inconsistent with cryo-ET findings, which reveal striking structural diversity among species [38–40,44–46,49]. In *Saccharomyces cerevisiae*, the densely packed nucleus is dominated by abundant protein complexes, with nucleosomes dispersed sparsely throughout the nucleoplasm [44]. By contrast, human Hela cells exhibit chromatin organised into large chromatin domains, where fibre-like conformations are largely absent [38,39]. Similarly, in human T lymphoblasts, nucleosomes predominantly aggregate within large chromatin domains, with chromatin fibres observed in heterochromatin regions near the nuclear envelop [40]. Chromatin in frog erythrocyte is presented as large domains and very distinctive zigzag helical fibres [49]. Intriguingly, the nuclei of green algae and higher plants lack the macromolecular crowding seen in other eukaryotes; instead, their chromatin exists as short, discrete fragments [45,55,56]. Beyond interspecies disparities, chromatin organisation is not static but dynamically remodels across stages of the cell cycle [46,47], further challenging the feasibility of a one-sizefits-all model.

## Key challenges in chromatin studies using cellular cryo-ET and cryo-FIB

Resolving the in-cell structure of nucleosomes at high resolution is critical for understanding chromatin organisation, yet significant technical hurdles persist. A single nucleosome, including its bound DNA, has a molecular weight of ~200 kDa [57]. Without the DNA, the histone octamer’s low density becomes nearly undetectable by visual inspection or template-matching algorithms, particularly within the crowded nuclear environment. While recent advances in cryo-ET have achieved in-cell ribosome structures at resolutions below 3 Å [58] and smaller complexes like Rubisco and TRiC at ~8 Å [59,60], these successes rely on either the large size or high symmetry of the particle. In contrast, mononucleosomes which are much smaller, asymmetric and embedded in dense chromatin present unique challenges.

The first major limitation is the inherently poor signal-to-noise ratio (SNR) of cellular tomograms compared to *in vitro* cryo-EM studies, further exacerbated by macromolecular crowding and technical constraints of cryo-FIB milling [37,61,62]. Current cryo-FIB protocols typically produce lamellae ~150 nm thick, rarely below 100 nm, which is the optimal thickness for 300 keV electrons [63]. Given a nucleosome’s dimensions (~11 nm), a 50 nm increase in lamella thickness introduces multiple overlapping nucleosome layers, degrading tomogram quality and complicating computational identification. This includes two major aspects: first, template-matching workflows, which normally employs low-pass-filtered nucleosome templates to locate individual nucleosome particles in 3D volumes, struggle with false positives due to noise from thick lamellae [37]; second, misaligned coordinates of nucleosome particles during the iterative refinement due to the low SNR and inaccurate initial matching results further hinder structural determination of nucleosomes in densely packed chromatin [37,64,65]. Therefore, *in vitro* cryo-ET with a reduced system and higher SNR also sees broader adoption in recent studies to elucidate mechanisms that require high-resolution information [49,51,53,66–68].

The second challenge lies in the heterogeneity of nucleosomes in the cell*. In vitro* cryo-EM achieves nearatomic resolution by purifying or reconstituting nucleosomes and acquiring images of millions of particles to sort conformational variability [21–24]. Cellular cryo-ET, however, lacks this luxury: STA prioritises dominant nucleosome conformations, relegating minority populations to ‘junk’ particles, and particles with subtle conformational changes are likely to be grouped into the same population, leaving many conformations of native nucleosomes structurally uncharacterised while eroding the resolution [44,46]. While limited resolution remains a key challenge in chromatin studies, low SNR and structural heterogeneity further compromise the accurate mapping of nucleosome within chromatin. As discussed above, only a subset of nucleosome particles is used for the final structural determination, and some nucleosome particles are omitted during template matching [44,46], fragmenting the broader structural context. This omission is particularly consequential, as chromatin architecture arises from the collective arrangement of individual nucleosomes; each unit’s position and orientation directly shape the three-dimensional morphology of the chromatin fibre. To unravel chromatin complexity, future cryo-ETefforts must prioritise advancements that simultaneously boost resolution and improve nucleosome coverage, ensuring a more comprehensive and precise representation of chromatin organisation.

Finally, cryo-ET workflows face practical limitations in both throughput and robustness. Sample loss and contamination risks arise at multiple stages: (1) during vitrification, where uneven cell distribution and grid damage from blotting are common; (2) during cryo-FIB milling, cellular lamellae could be bent and destroyed due to the deformation of supporting grids and scattering of the milling beam and (3) during lamella transfer from cryo-FIB to cryo-TEM, where handling introduces risks of ice contamination or lamella loss. These variables compound the difficulty of acquiring high-quality datasets at scale [37].

## Into the future of chromatin studies by cryo-ET and cryo-FIB

The future of chromatin studies through cryo-ET and cryo-FIB is poised for transformative breakthroughs. Despite existing challenges, advancements are being made, leading to determination of the in-cell structures of native nucleosomes and chromatin in much greater detail. Although nucleosomes are diminutive, their visualisation in high-quality tomograms has become feasible [40], allowing for precise localisation via enhanced template-matching algorithms. Innovations in cryo-FIB milling now explore plasma sources using light gases (e.g. argon, xenon) as alternatives to traditional gallium ions, which mitigate surface damage and yield more stable, high-quality cellular lamellae for imaging [69].

Computational strides, particularly in integration of artificial intelligence (AI) and machine learning into the current data analysis in cryo-ET and STA [70–73], can revolutionise in-cell chromatin analysis. These tools can enhance single-nucleosome detection in densely packed chromatin regions, reduce alignment biases and apply advanced denoising techniques to boost tomogram contrast [74,75], enabling clearer visualisation and segmentation. Moreover, the quality improvement of tomograms can drastically increase the coverage of individual nucleosomes in the chromatin, minimising the loss of structural context and revealing the highorder architecture of chromatin more precisely. Concurrently, improved and automated sample preparation workflows such as uninterrupted cryo-FIB lamella production [76] and high-throughput cryo-ET coupled with beam-shift acquisition [77], now generate hundreds of tilt series daily, minimising sample loss and accelerating data collection.

Moreover, utilising the cryo-correlative light and electron microscopy (cryo-CLEM), not only the structure of nucleosome and chromatin can be deciphered, but also pivotal transcriptional factors associated with various chromatin regions, as well as chromatin remodelling processes during cell cycles can be visualised in the native nucleus. In addition, a thin lamella only presents about 1% of the total cell volume with restricted field of view, combining *in vitro* and in-cell cryo-ETstudies with superresolution fluorescence microscopy, single-particle cryo-EM and canonical LLPS analysis [49,51,53,66–68,73,78–80] promises a holistic view of chromatin architecture, bridging nanometre- or even subnanometre-scale nucleosome details with larger domain organisation. Beyond model cell lines, extending these techniques to primary cells, tissues and disease or infection models will uncover chromatin variability across biological contexts. Furthermore, observations of nuclear condensates (e.g. nucleoli) are frequent in tomograms of intact nuclei [45]; however, the interaction between chromatin and nuclear condensates remains burgeoning area of research. Future cryo-ET studies could elucidate how phase separation governs chromatin dynamics and gene regulation in intact cells.

In summary, cryo-ET has emerged as a pivotal tool, overcoming the artefacts inherent in conventional EM and the resolution limits of fluorescence microscopy. It reveals chromatin as a flexible and phase-separated architecture intricately linked to functional states within native nuclei. As cryo-ET and cryo-FIB technologies advance, they hold immense potential to decode how chromatin’s physical structure encodes genomic logic, reshaping our understanding of epigenetics, cell cycle regulation, viral infection and disease mechanisms. This convergence of imaging innovations promises to unravel the deepest secrets of nuclear organisation, marking a new era in structural biology.

## Data Availability

No data was used for the research described in the article.
